# MK2 and Fas Receptor Contribute to the Severity of CNS Demyelination

**DOI:** 10.1371/journal.pone.0100363

**Published:** 2014-06-25

**Authors:** Silvia M. Tietz, Regina Hofmann, Tobias Thomas, Björn Tackenberg, Matthias Gaestel, Martin Berghoff

**Affiliations:** 1 Department of Neurology, Justus-Liebig-University Giessen, Giessen, Germany; 2 Theodor-Kocher-Institute, Universtiy of Bern, Bern, Switzerland; 3 Department of Child Neurology, Justus-Liebig-University Giessen, Giessen, Germany; 4 Department of Neurology, Clinical Neuroimmunology Group, Philipps-University, Marburg, Germany; 5 Institute of Biochemistry, Hannover Medical School, Hannover, Germany; Friedrich-Alexander University Erlangen, Germany

## Abstract

Models of inflammatory or degenerative diseases demonstrated that the protein-kinase MK2 is a key player in inflammation. In this study we examined the role of MK2 in MOG_35-55_-induced experimental autoimmune encephalomyelitis (EAE), the animal model for multiple sclerosis. In MK2-deficient (MK2^−/−^) mice we found a delayed onset of the disease and MK2^−/−^ mice did not recover until day 24 after EAE induction. At this day a higher number of leukocytes in the CNS of MK2^−/−^ mice was found. TNFα was not detectable in serum of MK2^−/−^ mice in any stage of EAE, while high TNFα levels were found at day 16 in wild-type mice. Further investigation revealed an increased expression of FasR mRNA in leukocytes isolated from CNS of wild-type mice but not in MK2^−/−^ mice, however *in vitro* stimulation of MK2^−/−^ splenocytes with rmTNFα induced the expression of FasR. In addition, immunocomplexes between the apoptosis inhibitor cFlip and the FasR adapter molecule FADD were only detected in splenocytes of MK2^−/−^ mice at day 24 after EAE induction. Moreover, the investigation of blood samples from relapsing-remitting multiple sclerosis patients revealed reduced FasR mRNA expression compared to healthy controls. Taken together, our data suggest that MK2 is a key regulatory inflammatory cytokines in EAE and multiple sclerosis. MK2^−/−^ mice showed a lack of TNFα and thus might not undergo TNFα-induced up-regulation of FasR. This may prevent autoreactive leukocytes from apoptosis and may led to prolonged disease activity. The findings indicate a key role of MK2 and FasR in the regulation and limitation of the immune response in the CNS.

## Introduction

The p38 mitogen-activated protein kinase (MAPK) pathway mediates cellular responses to injurious stress and immune signalling serving cell type-specific inflammatory functions that can result in cytokine and chemokine production. The downstream targets of p38 MAPK include the mitogen-activated protein kinase activated protein kinase (MK) 2 [1]. In response to cellular stress and cytokines, MK2 is activated by p38 in a phosphorylation dependent manner. Amongst the substrates of the p38 MAPK/MK2 pathway there are mRNA-AU-rich-element (ARE-)-binding proteins which regulate mRNA stability and translation of key inflammatory cytokines such as TNFα, IFNγ, IL-6 and IL-1β [1], [2]. Against this background MK2 is an interesting target in inflammation and has been studied in various disease models. In experimental asthma, MK2 *knockout* mice showed less airway inflammation because of a reduced vascular permeability of the blood-lung-barrier [3]. In models for Parkinson's disease and cerebral ischemia MK2-deficient mice showed reduced neurotoxicity and neuroinflammation [4], [5]. Moreover, after spinal cord injury reduced loss of neuronal cells and myelin was observed in MK2 knockout mutants [6]. These findings suggest that a lack of MK2 reduces inflammation and protects against destruction of brain cells and cells of other tissues.

Experimental autoimmune encephalomyelitis (EAE) is an inflammatory disease of the central nervous system (CNS), which serves as an animal model for multiple sclerosis (MS). Most characteristics of MS are reflected by myelin oligodendrocyte glycoprotein aa35–55 induced EAE: a chronic, relapsing clinical course of the disease, and a pathophysiological triad of inflammation, reactive gliosis, and formation of demyelinating plaques [7]. Inhibition of p38 was shown to weaken clinical symptoms of EAE [8], however, the role of MK2 in EAE is not yet defined. Given the fact that MK2 is a downstream target of p38 and acts as a key player in the regulation of the biosynthesis of pro-inflammatory cytokines we hypothesized that a lack of MK2 causes a less severe course of EAE, less CNS inflammation and brain destruction. To test this we induced EAE by MOG_35–55_ peptide in complete Freund's adjuvans in MK2^−/−^ mice. Contrary to our hypothesis, we observed that MK2^−/−^ mice showed a prolonged disease activity associated with an increased number of leukocytes in the CNS. A reason for these findings could be an inexistent up-regulation of the Fas-receptor mediated by TNFα in autoreactive leukocytes at the peak of EAE in MK2^−/−^ mice, resulting in prolonged disease activity and a loss of intrinsic limitation of the immune response by effector cell apoptosis.

More recently it has been suggested that activation induced cell death (AICD) is a key mechanism in the pathogenesis of MS and EAE [9], [10]. AICD is mediated by binding of Fas ligand (FasL) to the death receptor Fas (FasR, CD95), resulting in the intracellular recruitment of Fas associated death domain (FADD) and cleavage of caspase 8 [11]. A decreased expression of FasR was found in CD4+ CCR5+ T cells in relapsing-remitting (RR)-MS patients suggesting that the FasR contributes to the pathogenesis of MS by prolonging survival of autoreactive lymphocytes and enhancing migration of T cells into the CNS [12]. Therefore we investigated the FasR mRNA levels in sera from patients with RR-MS and correlated those findings with disability.

## Material and Methods

### Patients and controls

The investigation of human blood samples was approved by the local ethics committee for clinical research (University of Giessen No. 08/06). All patients provided their written informed consent to participate in the study prior to blood sampling. Blood samples were taken from 15 patients in relapse as well as three month later. The patients had no clinical relapses during at least 3 months before the blood sampling. Twelve out of 15 patients received intravenous methylprednisolone therapy for 3 to 5 days after blood sampling in relapse. A third group consisted of 15 RR-MS patients with no clinical activity for at least 5 to 9 month before the blood sampling. All patient groups were compared to 15 healthy individuals matched by gender and age [13] (Table 1). None of the patients received immunomodulatory drugs at the time of the study or three months before. All patients suffered from RR-MS according to the revised McDonald criteria [14] and had no other concomitant diseases.

**Table 1:** Demographic data of patients.

	relapse	remission	post remission	healthy donors
patients (females)	15 (12)	15 (12)	15 (12)	15 (12)
age (years) mean ± SD	38.9±7.4	38.9±7.4	39.0±7.4	38.8±7.2
disease duration (years) mean ± SD	3.7±4.7	3.7±4.7	3.4±4.4	-
EDSS mean ± SD	2.1±0.8	1.7±1.0	1.4±0.5	-
relapses within 2 years mean ± SD	0.6±0.6	0.6±0.6	0.8±0.7	-

Leukocytes were isolated from EDTA blood and lysates were stored at −70°C until total RNA was isolated using the QIAamp RNA Blood Mini Kit (Qiagen; Hilden, Germany).

### Mice

C57BL/6J wild-type mice were obtained from Harlan Winkelmann (Borchen, Germany). C57BL/6J ^Mapkapk2−/−^ (Mapkapk2 ^tm1Mg1^, MK2^−/−^) mice were described before [1]. Mice were housed in a room with controlled light cycle in the conventional animal husbandry of the Central Animal Laboratory Giessen. Age- and gender-matched (10 week-old female mice) wild-type mice and MK2^−/−^ mice were used in this study. All experiments were approved by local state authorities for animal studies (permission from the regional board Giessen, Hessen, Germany No. GI20/23-Nr.31 2008).

### Induction and clinical evaluation of MOG_35–55_ EAE

For EAE-induction, 300 µg of the MOG_35–55_ peptide (Charité Hospital, Berlin, Germany) in PBS emulsified with an equal amount of CFA containing 10 mg/mL mycobacterium tuberculosis H37RA (Difco, Augsburg, Germany) was injected subcutaneously at the four flanks. In addition mice received an intraperitoneal injection of 300 ng pertussis toxin [Calbiochem (Merck), Darmstadt, Germany] in PBS at the day of immunization and on day 2 after immunization. The clinical course of EAE was followed up until day 24 and the disease severity was assessed daily using the following score: 0, no clinical signs; 1, tail or hind limb weakness; 2, limp tail and hind limb weakness; 3, severe hind limb paresis; 4, complete hind limb paralysis and front limb weakness; 5 moribund.

### Isolation and evaluation of leukocytes from the spleen and from the CNS

Native brains and spinal cords were removed at day 8, day 16 and day 24 after EAE-induction, chopped in small pieces and digested with collagenase 1 mg/mL (GIBCO, Invitrogen corporation, Karlsruhe, Germany) and DNAse 30 U/mL (Qiagen, Hilden, Germany) for 60 min at 37°C. Mononuclear cells were purified by discontinuous percoll gradient of 1095–1030 g/mL and centrifugation for 30 min at 2,500 rpm [15]. Cells were washed twice and resuspended in 1X PBS. To confirm whether the cells are leukocytes cells were determined by flow cytometry (n = 3 each genotype). Cells were resuspended in FACS buffer (0.5% BSA, 0.02% NaN_3_ in 1X PBS). After Fc block incubation (1∶1000 for 15 min) cells were stained for either CD4 (ebioscience, San Diego, CA; 1∶100) or CD8 (ebioscience, San Diego, CA USA, 1: 100) and analysed using the FACSCalibur (BD GmbH, Heidelberg, Germany). Cell numbers were determined by microscopic inspection. Dead cells were excluded after staining with Trypan Blue. Decollating of splenocytes was performed using a 40 µm cell strainer. After centrifugation at 1,600 rpm for 4 min at 4°C erythrocytes were lysed in 10 mL lysis buffer (Qiagen, Hilden, Germany) for 10 min on ice. The cells were washed twice in 10 mL 1X PBS.

### Primary cell culture and stimulation

Isolated leukocytes from the spleen were pelleted (1400 rpm. for 4 min) and resuspended in RPMI 1640 medium (PAN-Biotec GmbH, Aidenbach, Germany) containing 10% FCS (PAN-Biotec GmbH, Aidenbach, Germany) and 1% antibiotics penicillin and streptomycin (GIBCO, Invitrogen corporation, Karlsruhe, Germany). Cells were maintained at 37°C in a humidified atmosphere of 5% CO_2_ and 95% air in 6-well-plates. Cells were stimulated with 50 ng/mL recombinant murine TNFα (ReliaTech, Wolfenbuettel, Germany) for 24 h.

### RNA-isolation, cDNA-synthesis and polymerase-chain-reaction (PCR)

Pelleted cells (splenocytes and leukocytes isolated from the whole CNS) were resuspended in 600 µL of RLT-buffer (Qiagen; Hilden, Germany). The RNA isolation was performed using the QIAamp RNA Blood Mini Kit (Qiagen; Hilden, Germany). The cDNA was synthesized from total RNA using RevertAid First Strand cDNA Synthesis Kit and oligo(dT) primers (Fermentas; St. Leon-Rot, Germany).

All PCR-primers were purchased from Eurofins MWG Operon (Ebersberg: Germany). FasR (CD95) mRNA levels was quantified by Real Time PCR with a SybrGreen Kit (Qiagen; Hilden, Germany) using the Light cycler system (Roche Diagnostics; Mannheim, Germany). PCR conditions for murine GAPDH was: 5 min at 95°C, then 45 cycles with 10 sec. at 95°C, 15 sec. at 60°C and 20 sec at 72°C (*fw* 5′-TGACGTGCCGCCTGGAGAAA-3′ and *rv* 5′-AGTGTAGCCCAAGATGCCCTTCAG-3′). PCR condition for murine FasR was: 5 min at 95°C, then 45 cycles with 10 sec at 95°C, 20 sec at 56°C and 20 sec at 72°C (*fw* 5′-CTGACCCAGAATACCAAG-3′ and *rv* 5′-AACAACCATAGGCCATTT-3′).

PCR conditions for human samples were: 5 min at 95°C, then 45 cycles with 10 sec at 95°C, 15 sec at 54°C (human Fas *fw* 5′-CGTCTGTTGCTAGATTATCG-3′ and *rv* 5′-TTGTCTGTGTACTCCTCCC-3′), respectively 60°C (human GAPDH: *fw* 5′-CCACATCGCTCAGACACCAT-3′; *rv* 5′-GGCAACAATATCCACTTTACCAGA-3′) and 20 sec at 72°C. To confirm specificity of the amplification a melting curve was generated by slowly increasing (0.1°C/sec) the temperature from 65°C to 95°C while fluorescence was measured. In each run probes were analyzed in duplicate. Finally all results were normalized to species according levels of GAPDH.

### Protein isolation and co-immunoprecipitation

Splenocytes were lysed with lysis buffer (150 mM NaCl, 20 mM Tris pH 7.4, 1 mM EDTA, 10% Glyzerin, 1% NP40, 0,01% NaN_3_, 20% β-Mercaptoethanol) containing protease inhibitor (Roche, Mannheim, Germany) for 1 h on ice. Cell debris was separated by centrifugation for 20 min at 13,000 rpm. For co-immunoprecipitation 40 µg of pooled protein samples (n = 3 each genotype) were filled up to a volume of 200 µL with ice-cold 1X cell lysis buffer (Cell Signaling; Frankfurt, Germany). Primary antibody α-cFlip was added 1∶50 and incubate with shaking overnight at 4°C. 20 µL of protein A agarose beads were added and incubated for 3 h at 4°C with shaking. Samples were centrifuged for 1 min at 14,000 rpm and washed with 500 µL cell lysis buffer (Cell Signaling; Frankfurt, Germany) for 5 times. The pellet was resuspended with 20 µl 3X SDS sample buffer +2 µL DTT. Samples were centrifuged for 1 min at 14,000 rpm and boiled for 5 min at 95°C. Separation of proteins was performed by 12% SDS-PAGE. Proteins were transferred onto a nitrocellulose membrane and incubated in blocking buffer (5% non-fat dry milk in TBST) for 1 h at room temperature. Primary antibody against FADD (Santa Cruz; Heidelberg, Germany) was incubated overnight at 4°C. After incubation of horseradish peroxidase-conjugated labeled IgG la antibody for 1 h at room temperature an enhanced chemiluminescence (ECL) solution (pierce; Rockford, IL USA) was used for immunodetection by the FusionFX7 (peqlab; Erlangen, Germany).

### Evaluation of TNFα levels

Blood was taken from healthy control and immunized mice (day 8, day 16 and day 24 after EAE-induction). Blood samples were allowed to clot for 2 h at room temperature. Afterwards samples were centrifuged for 20 min at 14,000 rpm and serum was pipetted in a fresh 1.5 mL tube. TNFα levels were evaluated by the Luminex MAgPIx system (Luminex/Invitrogen life technologies, Darmstadt, Germany) following the manufacturers protocol of the mouse cytokine magnetic 10-plex panel (Invitrogen life technologies, Darmstadt, Germany).

### Statistics

Statistical analysis was performed using GraphPad Prism 4 software. Differences between the groups during the single days in the course of EAE were evaluated using the unpaired student's *t* test.

Differences between mRNA expressions were analyzed by Kruskal-Wallis test. After detection of significant differences, post hoc analysis was performed using Mann-Whitney post hoc. The confidence interval for the analysis was 95% and statistical significance was set at *p*<0.05. The correlation analysis between the human Fas mRNA expression and the EDSS was performed using the Spearman's rank order correlation test.

## Results

### MK2-deficiency prolongs the disease activity of EAE

**Figure 1:**
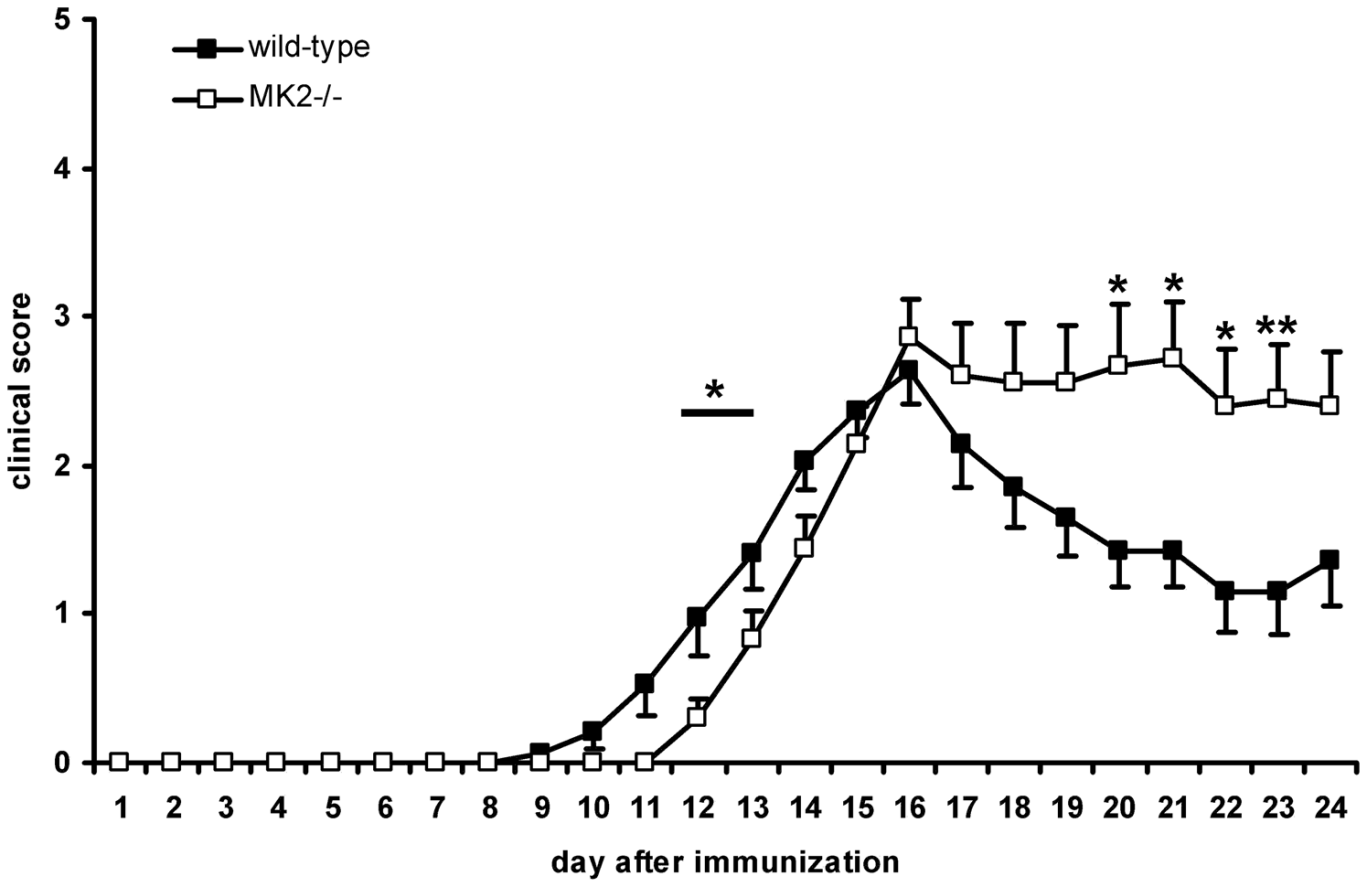
EAE-course in MK2^−/−^ mice compared to wild-type controls. EAE was induced actively with MOG_35–55_ in complete Freund's adjuvant in MK2^−/−^ mice and C57BL/6 wild-type control mice. The course of EAE was followed till day 24 post immunization and the signs of disease were assessed daily using a 5-point scale. Data are summarized from a total of three independent experiments. Only mice with EAE symptoms were included for the analysis of the clinical course (till day 8 n = 27 each genotype, till day 16 n = 18 MK2^−/−^; n = 15 wild-type, till day 24 n = 9 MK2^−/−^; n = 7 wild-type). MK2^−/−^ (open squares) mice showed a delayed onset of EAE (*p* = 0.0433) compared to wild-type mice (black squares). During the course of EAE both, MK2^−/−^ mice and wild-type mice developed progressive increasing signs of EAE with the same maximum score. After day 20 wild-type controls showed an improvement of the signs of EAE (went into remission) whereas MK2^−/−^ mice showed still severe symptoms of disease. Data are presented as mean ± SEM. *p<0.05; **p<0.001; students *t* test mean disease score from MK2^−/−^ mice compared to mean disease score of wild-type control mice at each single day.

Due to the fact that MK2 plays an important role in the biosynthesis of inflammatory cytokines it is an interesting target in autoimmune diseases. To gain insight into the function of MK2 in the pathogenesis of EAE, we actively immunized age- and gender-matched MK2^−/−^ mice and wild-type controls and studied disease development and activity. The clinical disease course was followed up until day 24 after EAE induction (Fig. 1). We found a delayed onset of EAE around day 13 in MK2^−/−^ mice compared to wild-type controls which developed clinical symptoms around day 12 post immunization (*p* = 0.0433) (Table 2). Both MK2^−/−^ and wild-type mice reached the peak of the disease at day 16. Over this period both MK2^−/−^ and wild-type mice developed a similar severity of the disease and the maximal score did also not differ (Table 2). Beyond the peak of EAE wild-type mice gradually recovered form EAE while the disease progressed in MK2^−/−^ mice. We found a significantly prolonged disease activity (*p*<0.05; *p*<0.001) in the remission that followed day 20 after immunization in MK2^−/−^ mice (Fig. 1).

**Table 2:** Basic data of EAE.

mice	incidence	mortality	day of onset (mean ± SEM)	severity of EAE day 16 p. i. (mean ± SEM)
C57BL/6	83%	0%	12.1±0.4	2.6±0.2
MK2^−/−^	100%	0%	13.1±0.2*	2.8±0.3

*p = 0.0433.

### Increased number of leukocytes inside the CNS of MK2^−/−^ mice

**Figure 2:**
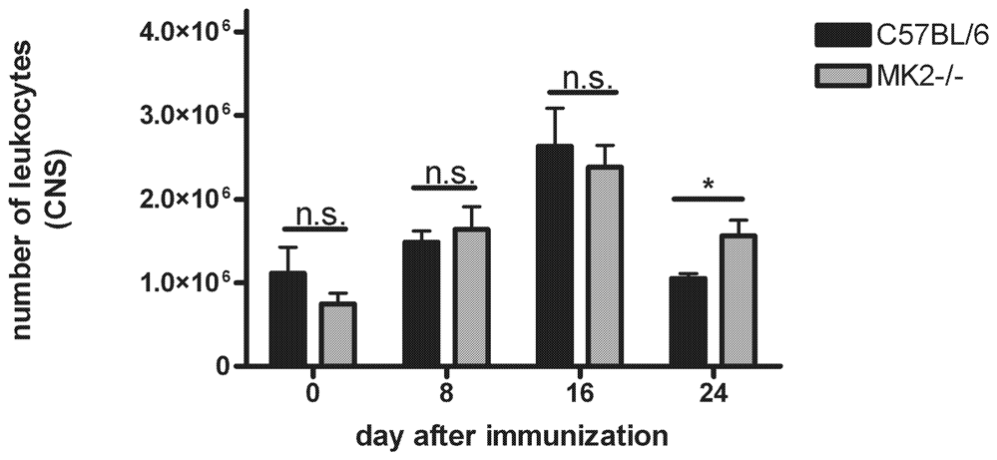
Numbers of infiltrating leukocytes in different stages of EAE. Infiltrating leukocytes were isolated from whole CNS (brain and spinal cord) from C57BL/6 wild-type control mice and MK2^−/−^ mice at different stages of EAE and cell numbers were determined using a Neubauer chamber. No difference in the number of infiltrated leukocytes was found at day 8 post immunization in wild-type (black bars) mice compared to MK2^−/−^ (grey bars) mice as well as at day 16. After the acute phase of EAE at day 24 post immunization less leukocytes were found in the CNS of wild-type mice compared to MK2^−/−^ mice (n = 6 each group). Data are presented as mean ± SEM. *p<0.05; students *t* test.

Then we asked why MK2^−/−^ mice showed a prolonged disease activity of EAE. We therefore evaluated the number of infiltrating leukocytes isolated from the CNS at different stages of EAE. Cells were isolated using a percoll gradient and leukocyte identity was verified by flow cytometry at day 16 and day 24 after immunization (n = 3 each genotype; *data not shown*). Both wild-type control mice and MK2^−/−^ mice showed successively increasing numbers of leukocytes to the peak of EAE (Fig. 2). In healthy controls approximately 1×10^6^ cells were found in wild-type mice and MK2^−/−^ mice. In the pre-clinical stage at day 8 around 1.5×10^6^ leukocytes were found in MK2^−/−^ mice and wild-type controls. The number of cells increased to a maximum of 2.5×10^6^ cells inside the CNS at the peak of the disease (day 16) in both groups. After the acute phase at day 24, however, the number of infiltrated leukocytes was decreased to approximately 1×10^6^ cells in wild-type controls whereas the numbers of CNS-infiltrating cells remained high in MK2^−/−^ mice (1.5×10^6^ cells; *p*<0.05).

### FasR mRNA expression is up-regulated at the peak of EAE in wild-type but not in MK2^−/−^ mice

**Figure 3:**
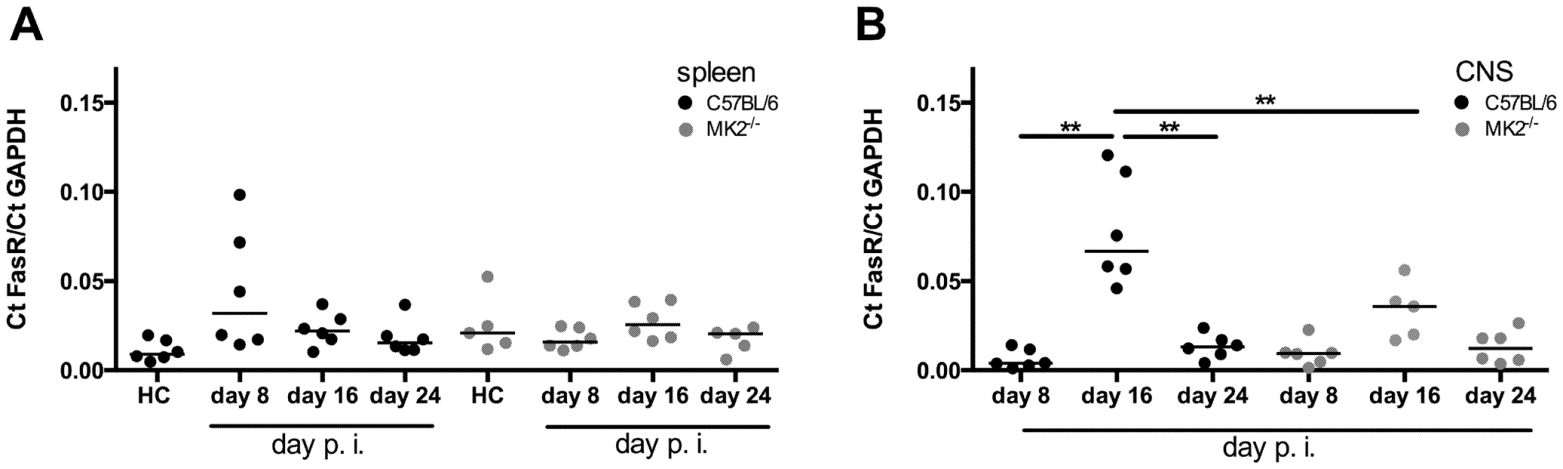
Fas-receptor mRNA expression in splenocytes and cerebral leukocytes in vivo. Splenocytes and cerebral leukocytes were isolated from C57BL/6 wild-type mice and MK2^−/−^ mice the FasR mRNA levels were determined. In splenocytes (A) there was no difference in the expression of FasR mRNA level observed between wild-type controls (black dots) and MK2^−/−^ mice (grey dots). In leukocytes isolated from CNS (B) there was an up-regulation of FasR mRNA level in wild-type controls (black dots) at day 16 after immunization. Each dot represents one single mouse. *Statistics*: non-parametric test (Kruskal-Wallis) and Mann-Whitney post *hoc* analysis; **p<0.001.

After we found a remained high number of leukocytes in the CNS of MK2^−/−^ mice at day 24 after EAE induction we requested the reason. A possible explanation for the remission of EAE is the apoptosis-induced cell death of inflammatory cells. To study whether the high number of leukocytes is caused by a lack of immune response limitation by apoptosis, we assayed the mRNA-expression of the death-receptors TNF receptor (TNFR) 1 and FasR in splenocytes and CNS-penetrated leukocytes at different stages of EAE (days 8, 16, and 24 after immunization) in wild-type controls and MK2^−/−^ mice. We did not observe any difference in the TNFR1-mRNA expression between wild-type mice and MK2^−/−^ mice in any stage of EAE studied, neither in splenocytes nor in leukocytes isolated from the CNS (*data not shown*). In accordance with these findings, no difference in the FasR-mRNA expression was found between splenocytes isolated from wild-type and MK2^−/−^ mice (Fig. 3A). We found no difference in the FasR-mRNA expression in splenocytes, neither in wild-type when compared to MK2-deficient mice nor at different time points studied. The FasR-mRNA in CNS-penetrated leukocytes isolated from wild-type controls was up-regulated at the peak of the disease (*p*<0.001) while there was no alteration in the FasR-mRNA expression in leukocytes isolated from the CNS from MK2^−/−^ mice (Fig. 3B).

### FasR mRNA expression is induced by TNFα

**Figure 4:**
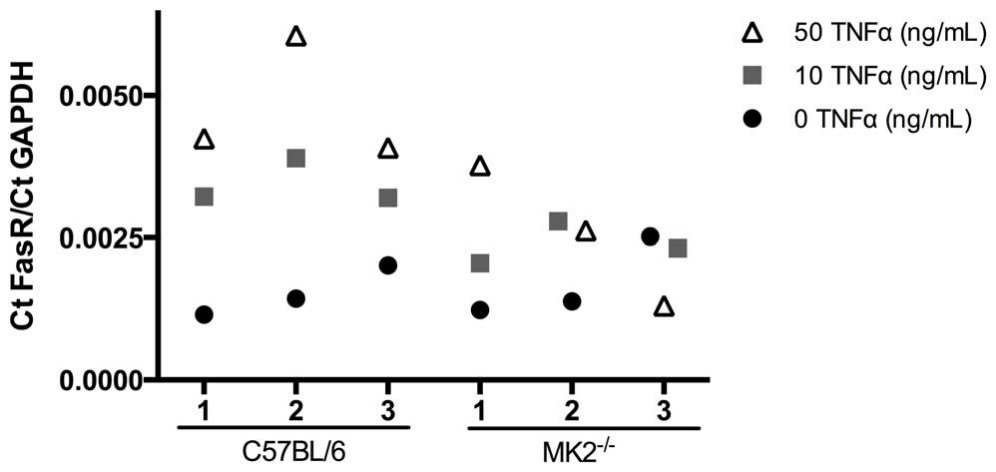
Fas-receptor mRNA expression in primary splenocytes in vitro. Splenocytes from healthy C57BL/6 wild-type mice and MK2^−/−^ mice were isolated and cultured *in vitro*. After stimulation with either 10 ng/mL (grey squares) or 50 ng/mL (open triangle) recombinant murine TNFα for 6 h the mRNA expression was analyzed by real-time PCR in comparison to untreated cells (black dots). The level of FasR mRNA was increased in wild-type mice after treatment with TNFα in a dose dependent manner (n = 3). Stimulation with 10 ng/mL TNFα of splenocytes obtained from MK2^−/−^ mice resulted in the up-regulation of FasR mRNA in mouse 1 and mouse 2. Higher dosage of TNFα (50 ng/mL) further increased FasR mRNA expression only in MK2^−/−^ mouse 1 but not in mouse 2. MK2^−/−^ mouse 3 showed opposed FasR mRNA levels.

Recent studies showed that the FasR mRNA expression as well as protein expression can be induced by TNFα [16], [17]. To investigate the contribution of TNFα to FasR mRNA expression we isolated primary splenocytes from healthy wild-type mice and MK2^−/−^ mice (n = 3) and stimulated them *in vitro* (Fig. 4). Semiquantitative real time-PCR revealed that FasR mRNA expression was increased in splenocytes after stimulation with 10 ng TNFα and further increased in response to 50 ng of TNFα compared to untreated cells isolated from wild-type mice. Furthermore, stimulation with 10 ng TNFα resulted in the same increase of FasR mRNA expression in two out of three MK2^−/−^ mice. An increased amount of TNFα stimulation up to 50 ng revealed inconclusive data in MK2^−/−^ mice. Mouse 1 showed exactly the same mRNA expression level like in splenocytes from wild-type while mouse 2 showed no further increased mRNA level. The mRNA expression of FasR in splenocytes isolated from MK2^−/−^ mouse 3 showed slightly decreased levels of the FasR mRNA.

### Decreased expression of TNFα in MK2^−/−^mice

**Figure 5:**
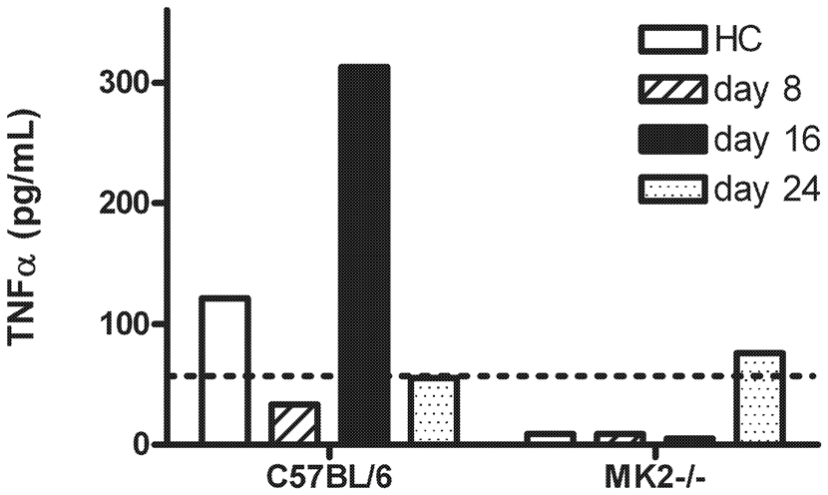
TNFα levels in serum of MK2^−/−^ mice and wild-type controls during EAE. TNFα levels were assayed in pooled serum from MK2^−/−^ and C57BL/6 wild-type controls (n = 9 each genotype) using the Luminex system during the course of EAE (day 8: striped bar; day 16: black bar; day 24: dotted bar) and healthy controls (white bar). The dashed line marks the detection limit. In wild-type controls a high amount of TNFα was found at the peak of the disease compared to the pre-clinical stage and the remission phase, while it was absent in MK2^−/−^ mice.

TNFα is expressed in an MK2-dependent manner [1]. We therefore investigated TNFα levels in serum in MK2^−/−^ mice and wild-type controls in all stages studied during EAE. The determination of TNFα in serum showed high levels of TNFα in the acute phase of EAE at day 16 in wild-type mice compared to both the pre-clinical stage of EAE at day 8 and after the acute phase at day 24, but was not detectable in MK2^−/−^ mice, indicating a correlation between high level of TNFα and up-regulated FasR mRNA expression in wild-type controls (Fig 5).

### Apoptosis of splenocytes is inhibited by cFlip in MK2^−/−^ mice

**Figure 6:**
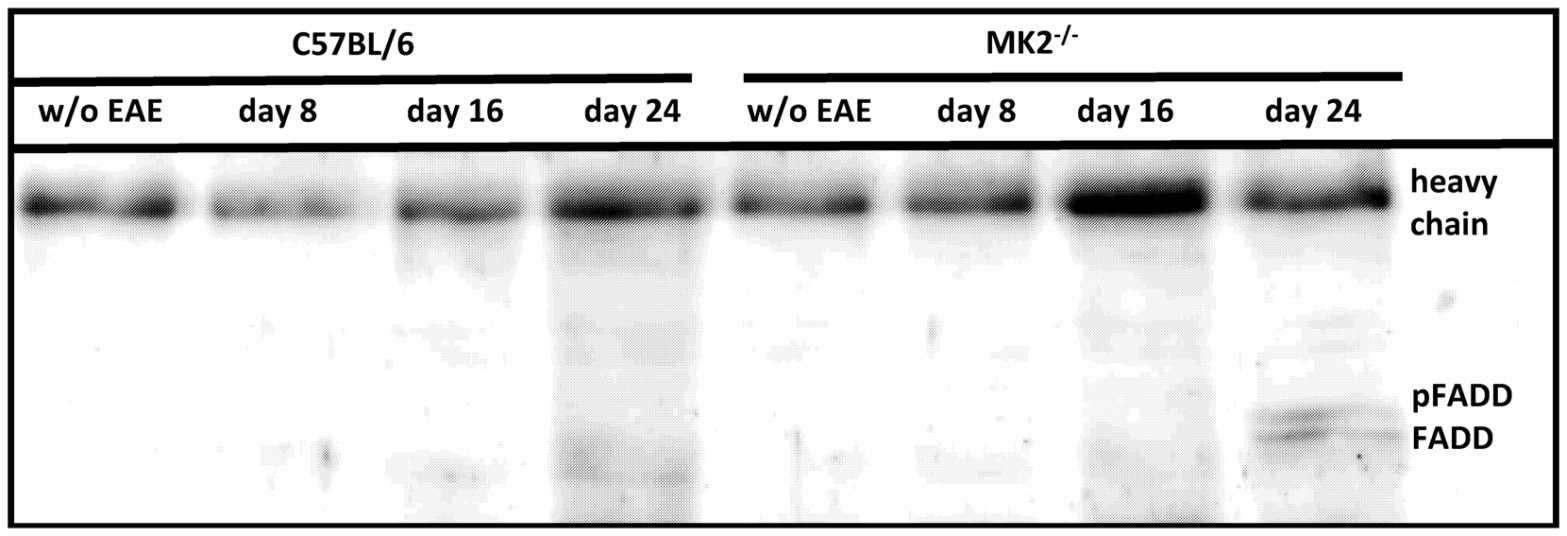
Evaluation of complex formation of cFlip and FADD by co-immunoprecipitation. Protein lysates of splenocytes were incubated with a cFlip antibody. Complexes between cFlip and associated proteins were isolated using protein A beads. The cFlip binding partner FADD was evaluated by western blotting. FADD was only detectable in MK2^−/−^ splenocytes at day 24 after immunization. Pooled samples (n = 3 each genotype) were used in three independent experiments. Fig. 6 shows a representative finding.

During acute EAE the frequency of autoaggressive T cells is high in the brain, but the vast majority of such cells continue to reside in the spleen [18], [19]. Elimination of autoreactive cells requires extrinsic apoptosis pathways such as CD95 signaling. FasR ligand-binding results in receptor the formation of death-inducing signalling complex through recruitment of the proteins FADD, procaspase-8 and cFlip. Processing of procaspase-8 at the DISC results in active caspase-8 inducing apoptosis. cFlip is able to inhibit apoptosis by competing with procaspase-8 for the binding site at the DISC [20], [21]. Against this background we investigated the cFlip-dependent inhibition of apoptosis in splenocytes by co-immunoprecipitation. We detected FADD and phospho-FADD at day 24 after immunization only in MK2-deficient mice but not in wild-type controls (Fig. 6).

### The Fas-receptor mRNA in patients with RRMS is decreased and correlates negatively with disabilities

**Figure 7:**
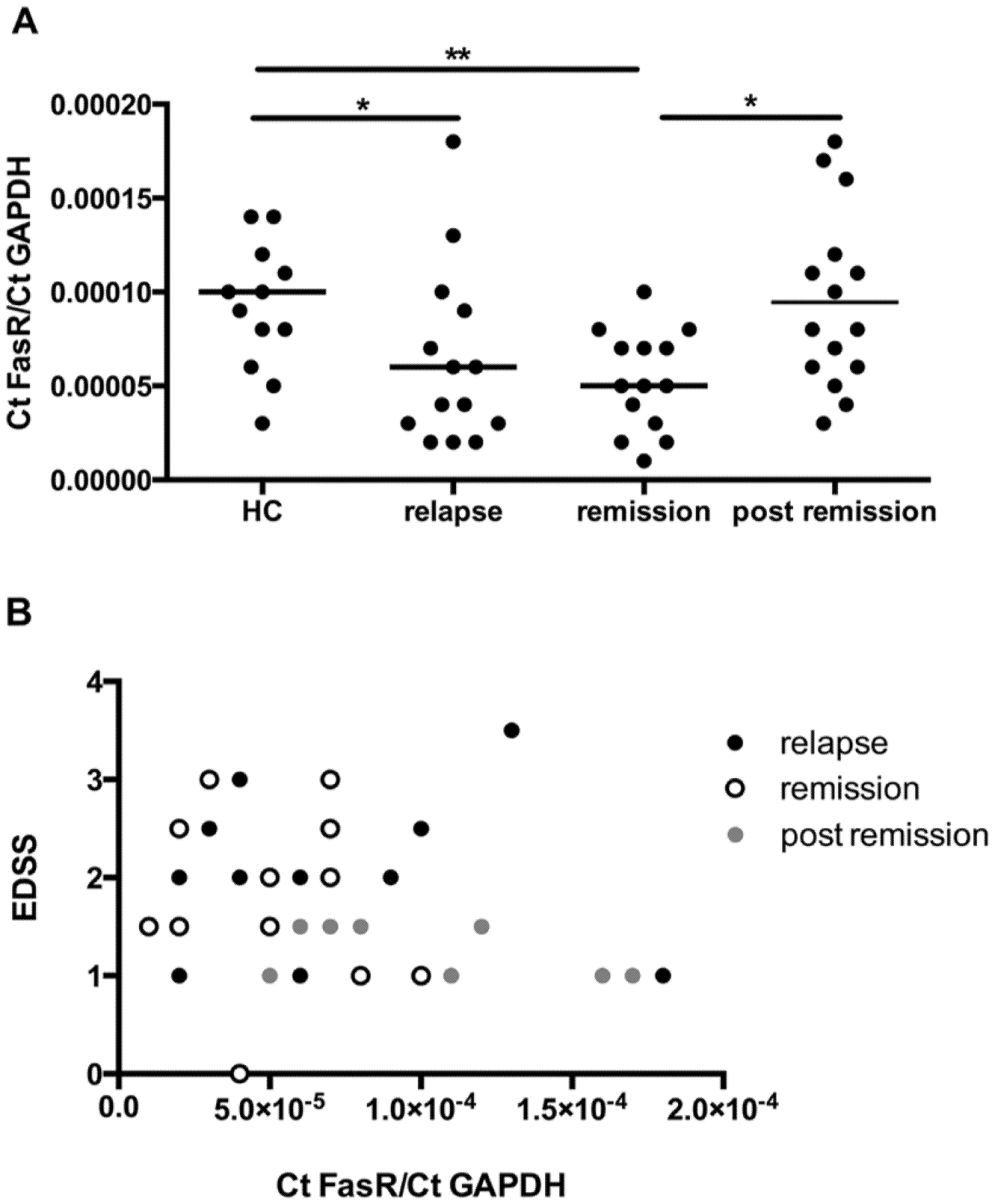
FasR mRNA expression in RR-MS patients and correlation to the EDSS. FasR mRNA expression in leukocytes obtained from patients with RR-MS and healthy controls (A). The analysis of the mRNA expression pattern of the FasR revealed a significant reduction of the mRNA in leukocytes from patients in relapse and three months afterwards compared to healthy donors. The comparison of FasR mRNA expression in leukocytes from RR-MS patients three months after relapse to five to nine months after relapse (post remission) showed a significant increase of FasR mRNA. No difference in FasR mRNA expression was observed between healthy donors and patients five to nine months after relapse (post remission). FasR mRNA expression in patients correlated to the EDSS (B). The interrelation analysis revealed a negative correlation between the EDSS and the FasR mRNA expression in all groups studied (*r* = −0,3061, *p* = 0,0409). Statistics: non-parametric test (Kruskal-Wallis) and Mann-Whitney post *hoc* analysis; *p<0.05, **p<0.001. The correlation analysis was performed using the Spearman's rank order correlation test.

After we found the decreased mRNA expression of FasR in leukocytes isolated from the CNS of MK2^−/−^ mice we studied whether patients with RR-MS show altered FasR mRNA levels in different stages of the disease. Therefore we assayed 15 blood samples from patients without immunomodulatory therapy in relapse as well as three month later in remission and compared these to 15 patient samples obtained five to nine months after relapse (post remission). All patients and healthy individuals were matched by gender and age (Fig. 7A; Table 1). The analysis of the mRNA expression pattern of the FasR revealed a significant reduction of mRNA in leukocytes from patients in relapse (*p*<0.05) and three months after in the remission (*p*<0.01) compared to samples obtained from healthy donors. Furthermore, the comparison of FasR mRNA expression in leukocytes from patients with RR-MS in remission and five to nine months afterwards (post remission) showed a significant increase of FasR mRNA (*p*<0.05). In addition we observed no difference in the FasR mRNA expression in leukocytes from healthy donors and patients five to nine months after relapse (post remission). To test whether these observations can be linked to disabilities we correlated the FasR mRNA expression to the EDSS (Fig. 7B). The interrelation analysis revealed a negative correlation between the EDSS and the FasR mRNA expression in all groups studied (*r* = −0.3061, *p* = 0.0409).

## Discussion

In this study we provide evidence for a role of the protein kinase MK2 in the course of EAE. Using MK2^−/−^ mice in the model system of MOG_35–55_-induced EAE, we demonstrated a prolonged course of the disease associated with a higher number of leukocytes in the CNS of MK2-deficient mice. Furthermore, the mRNA expression of the death receptor Fas was increased in leukocytes isolated from the CNS of wild-type controls at the peak of EAE while it remained unchanged in MK2^−/−^ mice. In correlation, we detected a high level of TNFα at the peak in wild-type controls while it was absent in MK2^−/−^ mice. This suggests a functional importance of MK2 and MK2-dependent cytokine production in limiting the immune response in EAE.

The role of MK2 was already investigated in various disease models. In all these studies using MK2^−/−^ mice the knockout was beneficial and mice showed ameliorated disease symptoms in e.g. experimental airway inflammation, spinal cord injury, Parkinson's disease and cerebral ischemia [3]–[6]. The role of MK2 in EAE, however, was not investigated before. But, the activation of its upstream kinase p38 was shown to be required for the development and progression of both chronic and relapsing-remitting EAE. Inhibition of p38 attenuated the signs of disease. More precisely, specifically the regulation of the p38 activity in T cells modulated the disease severity [8]. In addition, inhibition of p38α was shown to reduce the pathogenesis of EAE by decreasing the IL-17 production [22].

In contrast, we found that a lack of the downstream kinase MK2 was not an advantage in MOG_35–55_-induced EAE. The disease developed negatively in MK2^−/−^ mice showing a prolonged disease activity. This may indicate that other substrates of p38 beside the MK2 play a significant role in EAE. On the other hand, the outcome of p38 activation is not entirely clear. *In vitro* experiments using a fusion cytokine showed a hyperphosphorylation of p38 and amongst other pro-inflammatory cytokines, but also reduced the production of IL-17. Furthermore, the administration of this fusion cytokine in EAE mice suppressed symptomatic disease and correlated with decreased levels of inflammatory cytokines including IL-17, MOG-specific antibody titers, and blockade of CD4 and CD8 T cell infiltration in spinal cord [23].

Moreover, different models may account for the diverse courses of disease. The activation of p38 was inhibited in C57BL/6 mice by i.p. injection of the inhibitor SB203580 [8] a complete deletion of the downstream kinase MK2 was used in our study. In the present study we showed that elimination of MK2 in mice did not prove beneficial in EAE although the pro-inflammatory cytokine TNFα was reduced. But precisely this circumstance could be one reason for the prolonged disease activity in MK2^−/−^ mice.

TNFα is expressed in a MK2-dependent manner [1]. During the course of EAE TNFα was nonexistent in the serum of MK2^−/−^ mice while it was present at the peak of EAE in wild-type controls. Thus, MK2^−/−^ mice may resemble the course of disease of TNFα *knockout* mice. As in MK2^−/−^ mice used in our study, TNFα-deficient mice displayed a delayed disease onset compared to wild-type but led to an exacerbated form of EAE involving a prolonged disease activity in correlation with more mononuclear cells in the CNS [7], [24]–[26]. Moreover, anti-TNFα therapy in MS patients aggravated disease symptoms [27] and cell-type specific ablation of TNFα in T cells revealed exacerbated EAE [25]. The pathogenic and protective functions of TNFα in neuroinflammation were shown recently. Exogenous TNFα exacerbated focal ischemic injury and blocking of endogenous TNFα was neuroprotective [28], [29]. In contrast in mice lacking the TNFR1 neuronal damage after focal cerebral ischemia-reperfusion was significantly increased suggesting a neuroprotective role of TNFα after acute brain insults [29]. This encourages the assumption that a lack of the neuroprotective function of TNFα may result in the more severe chronic EAE in MK2^−/−^ mice.

One protective function of TNFα might be the limitation of an immune response by TNFα-induced up-regulation of the FasR. Certainly, the role of FasR in EAE is controversial. On the one hand, mice lacking the FasR showed milder and no EAE signs compared to wild-type animals, respectively [30]. Other studies showed a more severe course of disease in FasR-deficient mice while FasR-deficient SJL mice generally failed to remit from the acute clinical disease [31], [32]. However, the FasR seems to be a key mediator in the induction of apoptosis in CD4^+^ T cells [33]. Like in our study, in SJL mice the FasR mRNA expression reached a maximum level in mice undergoing acute clinical disease. Then it gradually declined to low levels in remission, suggesting an important role of FasR in the spontaneous recovery from EAE [31], [32]. Moreover, inhibition of p38 MAPK by the specific inhibitor SB203580 prevents AICD [34].

In our study in wild-type mice the FasR mRNA was up-regulated at the peak of EAE in leukocytes isolated from CNS as well as TNFα in serum. In contrast, no TNFα was present in serum of MK2^−/−^ mice during EAE and also no alteration of the FasR mRNA expression was found in leukocytes from the CNS. In this sphere it could mean for instance, that effector T cell apoptosis is reduced in MK2^−/−^ mice because of a lacking TNFα-induced up-regulation of FasR leading to the increased number of leukocytes and acute inflammation in the chronic phase of EAE.

Moreover, cFlip is believed to be an important modulator of apoptosis [35]. During EAE autoreactive leukocytes are continuously recruited from the peripheral organs such as spleen [19]. The analysis of splenocytes at day 24 after EAE induction showed complex formation between cFlip and FADD only in MK2^−/−^ mice. These data suggest that apoptosis of autoimmune cells is actively inhibited in the spleen of MK2-deficient mice.

In order to characterize the lack of up-regulation of the FasR mRNA in leukocytes isolated from MK2^−/−^ mice we found a negative correlation between Fas mRNA and caspase-8 mRNA (*data not shown*) in leukocytes isolated from RR-MS patients and disability. Low levels of Fas mRNA were associated with higher disability. Previously it has been reported that FasR mRNA is up-regulated in MS patients [36], [37]. It has also been shown that T cells from MS patients with are more resistant to apoptosis mediated by the FasR than cells from healthy individuals [9], [38], [39]. Differences between our data and these studies quoted above including time points of sample taking, medication of patients and demographic data may account for this. In our study we used an approach to analyse patients at two time points without clinical activity but possibly immunological activation and in relapse, where changes in inflammation are known to take place both peripherally [40] and in the CNS [41], [42]. Apoptosis of leukocytes is a key element in the regulation of a chronic immune response and is mediated by death receptors and depending signalling transduction cascades [11]. Particularly FasR is an important mediator of AICD, which is needed for the elimination of T cells during a chronic immune response [34]. A reduction of Fas mRNA levels in relapse and remission suggests that apoptosis is impaired and that reduced apoptosis is sustained for months. These data provide evidence for an immunological process, in which leukocytes escape cell death, prolong survival and sustain inflammation.

## Conclusions

In summary, the present study underlines the significance of MK2 in the regulation of CNS inflammatory diseases such as the multiple sclerosis animal model EAE. In contrast to the initial hypothesis, the MK2 deficiency aggravated the course of disease and was associated with more cellular CNS inflammation. These findings may be explained by a deficiency of key inflammatory cytokines leading to a lack of apoptosis of autoreactive leukocytes and thus to a missing limitation of the immune response in the CNS of MK2^−/−^ mice. In addition, low levels of FasR mRNA in patients with RR-MS are associated with impaired disability. These data in humans provide evidence for a FasR mediated limitation of the immune response essential for the recovery from relapse.
